# Supplementary material for: Social networks influence farming practices and agrarian sustainability
Source: PLoS One. 2021 Jan 7;16(1):e0244619. doi: 10.1371/journal.pone.0244619 (PMC7790232; doi:10.1371/journal.pone.0244619)
Supplement: S1 File — (DOCX) [file pone.0244619.s001.docx]

# Farmers and landholders survey

The time required to complete the survey: 30 minutes

This is to invite you to participate in a study entitled “Social capital in agrarian systems networks” which is being conducted by Dr. Amaia Albizua. This work is a collaboration of the Basque Centre for Climate Change and McGill University (department of sustainability), with funding from the Basque Government. The purpose of this research is to investigate local agrarian networks.

Many farmers and rural owners have a network of other farmers and people connected to the farming sector with whom they share information and talk about seeds, fertilisers, alternatives to fight plagues, crop rotations, irrigation, and other farming management topics. Sometimes these conversations with others just pass the time and it is enjoyable to talk story, and sometimes they provide valuable information that can contribute to farming performance.

We're interested in knowing a little about you and who is in your network of farmers. First, we’d like to ask a few questions about you and your experience with farming. Second, we will explore your network to understand who influences you when making decisions about land management and your farm performance and sustainability.

Your participation in the study will entail an oral survey, lasting approximately 30 minutes. If you have any questions regarding the project or questionnaire, please let me know now.

Your participation in the questionnaire is voluntary. The personal information you provide us will be confidential, no names will appear later in future scientific publications or oral presentations. All data collected will be stored by the main researcher (myself) at McGill University during 2018 and the Basque Center for Climate Change (BC3) during 2019, following the privacy policies of the university and research center respectively. All identifiable paper materials will be kept in a locked location only accessible to myself. All electronic materials will be kept on an encrypted file on my password-protected computer.

There are no expected risks for the participants from this research. In contrast, there is an opportunity for knowledge sharing among academics and rural sector personnel, government managers, and private landowners, as well as better understanding the ecological and social drivers of agrarian ecosystem services co-production.

I would like to audio-tape the survey. This will be only to ensure accuracy and the tape will be destroyed once the interview has been transcribed. When you are ready to begin, continue to the next section of the questionnaire.

*Compulsory

Code / Date

Participants characteristics

1. Name
2. Nickname
3. Age
4. Gender
5. Farming experience (years)

Land management. Please, let me know:

1. The total surface you labor (Ha)
2. Crops (list them)
3. Fertilizer type you use (mineral, organic, mix)
4. Irrigation type (sprinkler, drop, flood, none)

Involvement in decisions for farming:

1. Farming practices
2. Hiring of human labour
3. Technology pursues and maintenance

Network information. Now we would like to learn more about your network and explore with you how your network may help you with farming or influence your decisions about land management and farming practices. We’d like you to begin by identifying up to five with whom you exchange knowledge/information (regularly or punctually but important for you) with or get advice from about farming, and then we’d like to learn a little more about each of them. Please, you could start with the person you probably talk to the most and we can go on from there.

1. Person 1name and surname
2. Person 1 category does that person belong (farmer, family, cooperative, agrarian union (specify which), government advisor, private enterprise (specify which), others)
3. Type of knowledge exchange (open question, followed later by a list of topics generated in the trail)
   1. Crop varieties
   2. Crop rotations alternatives
   3. Communal lands
   4. Water access policies
   5. Seeds
   6. Irrigation water management
   7. Fertilisers
   8. Herbicides
   9. Water prices
   10. Agrarian subsidies
   11. Agrarian insurances
   12. Agrarian discounts
   13. Finance advice
   14. Crop prices
   15. Policies and norms
   16. Technological advances
   17. Technological maintenance
   18. Meteorological forecasts
   19. Crop illnesses
   20. Land erosion
   21. Others
4. The frequency you talk to Person 1 (Not often (1-3 times/year), Sometimes (1-3 times/month), very frequently (1-3 times/week or more))
5. The importance you attach that such conversations (not important, a bit important, important, very important)
6. The reason you relate to Person 1 (only one option selection)
   1. Altruism
   2. Dependency
   3. Duty
   4. Common interest
   5. Laboral

This was repeated 5 times or the number of names the participant had mentioned.

1. Do you think the way you are connected helps to achieve your farming goals? (yes, no, don’t know)
2. Which ecosystem services do you think you enhance through the way you manage your land?
   1. Food
   2. Biodiversity
   3. Fertility
   4. Habitat
   5. Water regulation (quality and quantity)
   6. Climate regulation
   7. Plagues regulation
   8. Land pollution absorption
   9. Soil erosion
   10. Education
   11. Traditions
   12. Landscape
   13. Traditional knowledge

# Organizations interviews

Code / Date

Participants attributes

1. Name
2. Age
3. Gender
4. Type of organization
5. The role played in the organization
6. Working experience (years)
7. Decisions made within the organization

Organization role affecting farmers’ land management decision-making. Please, let me know:

1. What are the main goals/duties of your organization
2. How, on what issues, and to what extent do you think your organization can affect farmers’ decision-making

Organization’s connections

1. Mention up to five other organizations you relate with to perform your activity
2. Person 1 category
3. Type of knowledge exchange (open question)
4. The frequency you talk to Person 1 (Not often (1-3 times/year), Sometimes (1-3 times/month), very frequently (1-3 times/week or more))
5. The importance you attach that such conversations (not important, a bit important, important, very important)
6. The reason you relate to Person 1

This was repeated 5 times or the number of names the participant had mentioned.

*Those organizations connections are not included in the network analysis but were helpful to identify others that could be interviewed to further understanding how farmers are influenced by other stakeholders outside their community.
