# Supplementary material for: Social networks influence farming practices and agrarian sustainability
Source: PLoS One. 2021 Jan 7;16(1):e0244619. doi: 10.1371/journal.pone.0244619 (PMC7790232; doi:10.1371/journal.pone.0244619)
Supplement: S1 Table — (DOCX) [file pone.0244619.s002.docx]

# Hierarchical cluster analysis table results

Table A contains information about the class, mode, p-value, and v-test, and allow the interpretation of the performed HCAs. To understand HCA results, we first selected only the variables with a p-value lower than 0.05 (less than 5%), as per the variables in Table A. This means that the categories within a given variable were significantly different among the farmers’ groups in 95% of the cases. In this way, each cluster is represented by some variables that are significantly different across farmers’ groups. For example, if we look at the first cluster and the first row of Table A, we can infer that the first cluster, intensive farmers, is linked to the category of using sprinkler irrigation and cultivate maize and biofuels mixing organic and mineral fertilizers in large surfaces -between 50 and 100 Has and more than 100 Has. This can be explained by looking at the value of Cla/Mod, which reveals that 100% of farmers cultivating surface between 50 and 100 Has and more than 100 Has belonged to the cluster of intensive farmers. Thus, this information explains that the farmers laboring large-scale and cultivating maize and biofuels are overrepresented in this cluster.

**S1 Table Characterization of one of the clusters regarding farmers’ land use management (N=45)**

|  | Cla/Mod | Mod/Cla | Global | p.value | v.test |
| --- | --- | --- | --- | --- | --- |
| fsprinkler=fsprinkler_yes | 76.27 | 100.00 | 72.84 | 0.00 | 6.46 |
| fmaize=fmaize_yes | 81.63 | 88.89 | 60.49 | 0.00 | 5.91 |
| fmixed=fmixed_yes | 87.50 | 62.22 | 39.51 | 0.00 | 4.75 |
| fbiofuel=fbiofuel_yes | 92.31 | 53.33 | 32.10 | 0.00 | 4.72 |
| forganic=forganic_yes | 79.49 | 68.89 | 48.15 | 0.00 | 4.16 |
| fmineral=fmineral_yes | 69.64 | 86.67 | 69.14 | 0.00 | 3.76 |
| fCultivated_Area2=50-100Ha | 100.00 | 24.44 | 13.58 | 0.00 | 3.34 |
| fdropping=fdropping_no | 71.74 | 73.33 | 56.79 | 0.00 | 3.31 |
| fcereal=fcereal_yes | 64.62 | 93.33 | 80.25 | 0.00 | 3.24 |
| fCultivated_Area2=>100Ha | 100.00 | 17.78 | 9.88 | 0.01 | 2.71 |
| fgrass=fgrass_yes | 77.78 | 31.11 | 22.22 | 0.03 | 2.12 |
| fCultivated_Area2=5-10Ha | 73.91 | 37.78 | 28.40 | 0.04 | 2.06 |
| fvineyard=fvineyard_yes | 75.00 | 33.33 | 24.69 | 0.05 | 1.98 |
| fothers=fothers_no | 65.22 | 66.67 | 56.79 | 0.05 | 1.96 |
| fvineyard=fvineyard_no | 49.18 | 66.67 | 75.31 | 0.05 | -1.98 |
| fgrass=fgrass_no | 49.21 | 68.89 | 77.78 | 0.03 | -2.12 |
| fothers=fothers_yes | 41.18 | 31.11 | 41.98 | 0.03 | -2.17 |
| fcereal=fcereal_no | 18.75 | 6.67 | 19.75 | 0.00 | -3.24 |
| fdropping=fdropping.NA | 0.00 | 0.00 | 12.35 | 0.00 | -3.82 |
| fsprinkler=fsprinkler.NA | 0.00 | 0.00 | 12.35 | 0.00 | -3.82 |
| fsprinkler=fsprinkler_no | 0.00 | 0.00 | 14.81 | 0.00 | -4.29 |
| fbiofuel=fbiofuel_no | 38.18 | 46.67 | 67.90 | 0.00 | -4.72 |
| fmixed=fmixed.NA | 0.00 | 0.00 | 18.52 | 0.00 | -4.97 |
| forganic=forganic.NA | 0.00 | 0.00 | 18.52 | 0.00 | -4.97 |
| fmineral=fmineral.NA | 0.00 | 0.00 | 18.52 | 0.00 | -4.97 |
| fmaize=fmaize_no | 15.63 | 11.11 | 39.51 | 0.00 | -5.91 |
| fCultivated_Area2=0-5Ha | 3.70 | 2.22 | 33.33 | 0.00 | -6.90 |
