# Supplementary material for: Social networks influence farming practices and agrarian sustainability
Source: PLoS One. 2021 Jan 7;16(1):e0244619. doi: 10.1371/journal.pone.0244619 (PMC7790232; doi:10.1371/journal.pone.0244619)
Supplement: S2 Table — (DOCX) [file pone.0244619.s003.docx]

# Attempt to include environmental variables

Environmental variables were extracted from the Geographical Information System (GIS)-based maps (see Table B). We used 1:25000 scale available maps downloaded from https://idena.navarra.es/navegar. Although the climate is determinant for farming decision-making, it is not included since all plots are in a range of 20 km and we consider climate does not change much in this range.

**S2 Table. Environmental variables considered being included in the model**

| **Drivers** | **Definition** | **Select examples** | **Evidence in rural communities** | **Contextually relevant**  **drivers** |
| --- | --- | --- | --- | --- |
| Exogenous contextual factors | Cultural, social, geographic, and/or ecological environments of individual actors drive the establishment and maintenance of social ties | Geography and space brings and holds people together (Rivera et al. 2010; Lusher et al. 2012) | Fishing grounds (e.g. Maya-Jariego et al. 2016)*  Landing sites (no empirical work to date) | Geographic Proximity  Lithology  Habitat conservation  Fire affection  Potential vegetation  Hunting area  Aquifers pollution risks |

We did not finally add those variables because we only have data for around half of the participants (N=48). This occurred because, during the fieldwork, many farmers denied to point where their plots were on a map. Some preferred not to reveal it and others just couldn’t find their plots in this way. This high degree of missing data provides noise to the models. Besides, one person normally had more than one plot, which provided us a big range of data.
