# Supplementary material for: Social networks influence farming practices and agrarian sustainability
Source: PLoS One. 2021 Jan 7;16(1):e0244619. doi: 10.1371/journal.pone.0244619 (PMC7790232; doi:10.1371/journal.pone.0244619)
Supplement: S3 Table — (DOCX) [file pone.0244619.s004.docx]

# S3 Table. Land management differences among the groups of farmers with NCP co-production awareness

|  | **Mean value of farmers within the group aware of their negative impacts (N=19)** | **Mean value of farmers within climate regulators and landscape advocates group (N=42)** | **Mean value of farmers within the traditionalist and habitat supporters group(N=20)** | **p-value** | **corrected**  **p-value** |
| --- | --- | --- | --- | --- | --- |
| 0-5 Ha cultivated land | 0.11 | 0.23 | 0.35 | 0.07 | 0.38 |
| 5-50 Ha cultivated land | 0.26 | 0.38 | 0.35 | 0.59 | 0.80 |
| 50-100 Ha cultivated land | 0.16 | 0.08 | 0.13 | 0.83 | 0.80 |
| 100-300 Ha cultivated land | 0.26 | 0.23 | 0.13 | 0.28 | 0.72 |
| >300 Ha cultivated land | 0.21 | 0.08 | 0.04 | 0.09 | 0.38 |
| Cereal | 1.00 | 0.85 | 0.70 | 0.01** | 0.13 |
| Biofuel | 0.53 | 0.38 | 0.35 | 0.26 | 0.72 |
| Vineyard | 0.26 | 0.38 | 0.26 | 0.94 | 0.80 |
| Grass | 0.26 | 0.08 | 0.35 | 0.46 | 0.38 |
| Other crops  (vegetables and fruit trees) | 0.21 | 0.62 | 0.57 | 0.03* | 0.13 |
| Maize | 0.79 | 0.69 | 0.52 | 0.07 | 0.38 |
| Mineral fertilizer | 0.95 | 0.92 | 0.74 | 0.05* | 0.38 |
| Organic fertilizer | 0.53 | 0.62 | 0.74 | 0.15 | 0.58 |
| Mixed fertilizer | 0.47 | 0.62 | 0.57 | 0.59 | 0.80 |
| Sprinkler irrigation | 1.00 | 0.85 | 0.70 | 0.01** | 0.13 |
| Dropping irrigation | 0.16 | 0.62 | 0.57 | 0.01** | 0.13 |

‘*’ *p* < .05; ‘**’ *p* < .01 Kruskal Wallis test.

The corrected p-value refers to adjustment for multiple comparisons using the False Discovery Rate.
