# Supplementary material for: Social networks influence farming practices and agrarian sustainability
Source: PLoS One. 2021 Jan 7;16(1):e0244619. doi: 10.1371/journal.pone.0244619 (PMC7790232; doi:10.1371/journal.pone.0244619)
Supplement: S4 Table — (DOCX) [file pone.0244619.s005.docx]

**S4 Table. ERGM to check homophily in the type of farming effect in ties formation**

| Summary of model fit | | |  |  |  |  |
| --- | --- | --- | --- | --- | --- | --- |
| ========================== | | | |  |  |  |
| Formula: nedge.general ~ edges + nodematch("clust_manag", diff = T) | | | | | | |
| Iterations: 6 out of 20 | | |  |  |  |  |
|  |  |  |  |  |  |  |
| Monte Carlo MLE Results: | | | | | | |
|  | Estimate Std. | Error | MCMC % | z value | Pr(>\|z\|) |  |
| edges | -3.40273 | 0.08915 | 0 | -38.17 | <1e-04 | *** |
| nodematch.clust_manag.1 | 0.38843 | 0.27114 | 0 | 1.433 | 0.152 |  |
| nodematch.clust_manag.2 | -0.33612 | 0.18312 | 0 | -1.836 | 0.0664 | . |
| nodematch.clust_manag.3 | -0.72976 | 0.42103 | 0 | -1.733 | 0.083 | . |
| Signif. Codes | 0 | ‘***’ | 0.001 | ‘**’ | 0.01 | ‘*’ |
| AIC: | 1728 | BIC: | 1755 | (Smaller | is | better.) |
