# Supplementary material for: Social networks influence farming practices and agrarian sustainability
Source: PLoS One. 2021 Jan 7;16(1):e0244619. doi: 10.1371/journal.pone.0244619 (PMC7790232; doi:10.1371/journal.pone.0244619)
Supplement: S5 Table — (DOCX) [file pone.0244619.s006.docx]

**S5 Table. ERGM to check to belong to uneven types of farming effect in ties formation**

| Summary of model fit | | |  |  |  |  |
| --- | --- | --- | --- | --- | --- | --- |
| ========================== | | | |  |  |  |
| Formula: nedge.general ~ edges + nodefactor("clust_manag") | | | | | | |
| Iterations: 6 out of 20 | | |  |  |  |  |
|  |  |  |  |  |  |  |
| Monte Carlo MLE Results: | | | | | | |
|  | Estimate Std. | Error | MCMC % | z value | Pr(>\|z\|) |  |
| edges | -2.8161 | 0.1695 | 0 | -16.611 | 1.00E-04 | *** |
| nodefactor.clust_manag.2 | -0.4191 | 0.1194 | 0 | -3.509 | 0.00045 | *** |
| nodefactor.clust_manag.3 | -0.5668 | 0.1492 | 0 | -3.8 | 0.000145 | *** |
| Signif. Codes | 0 | ‘***’ | 0.001 | ‘**’ | 0.01 | ‘*’ |
| AIC: | 1718 | BIC: | 1738 | (Smaller | is | better.) |
