# Supplementary material for: Social networks influence farming practices and agrarian sustainability
Source: PLoS One. 2021 Jan 7;16(1):e0244619. doi: 10.1371/journal.pone.0244619 (PMC7790232; doi:10.1371/journal.pone.0244619)
Supplement: Attachment — Submitted filename: PONE-D-20-24320_reviewer.pdf [file pone.0244619.s008.pdf]

PLOS ONE

What affects farmers’ advice networks: implications for agrarian sustainability

--Manuscript Draft--

**Manuscript Number:**	PONE-D-20-24320
**Article Type:**	Research Article
**Full Title:**	What affects farmers’ advice networks: implications for agrarian sustainability
**Short Title:**	What affects farmers’ advice networks
**Corresponding Author:**	Amaia Albizua<br>Basque Centre for Climate Change BC·<br>Leioa, Bizkaia SPAIN
**Keywords:**	social network structure, social network analysis, agriculture intensification, sustainability
**Abstract:**	Agriculture intensification, and its ecological and social effects, is pushing researchers to seek a rural transformation that supports biodiversity and agrarian ecosystems' functions. Social networks researchers have established that networks influence and are influenced by individuals' thoughts, values, and behaviors. However, there is incomplete knowledge about the factors that influence farmers’ connections to seek advice about land management, and few studies addressing farmers’ perceptions about their influence on agrarian ecosystems. We use interviews and social network data in a farming community undergoing accelerated agricultural intensification in Navarre, Spain to explore 1) farmers’ perceptions regarding the effects of their management on the agrarian ecosystem and 2) the factors driving farmers’ land management. We found that intensive farmers acknowledge their practices’ negative effects on the agrarian ecosystem, and small-scale farmers recognize their contribution to traditions, traditional knowledge, landscape, and their capacity to regulate the climate. However, farmers negatively affecting the ecosystems control the information flow in our studied rural network whereas farmers aware of their contribution to climate regulation ask more for advice from other groups of farmers while they are not frequently asked for advice. Those results inform rural policies about how and why intensive practices dominate over sustainable practices and uncover that incentivizing small-scale farmers' participation in the advice network could decrease the current obstacles for farmers’ engagement with sustainable farming practices.
**Order of Authors:**	Amaia Albizua
	Elena Bennett
	Guillaume Larocque
	Robert Krause
**Additional Information:**	
**Question**	**Response**
**Financial Disclosure**<br><br>Enter a financial disclosure statement that describes the sources of funding for the work included in this submission. Review the submission guidelines for detailed requirements. View published research articles from *PLOS ONE* for specific examples.<br><br>This statement is required for submission and **will appear in the published article** if the submission is accepted. Please make	AA, Eusko Jaurlaritza(Postdoctoral Scholarship); https://www.euskadi.eus/informacion/ayudas-al-personal-investigador-programa-posdoctoral/web01-a2hunib/es/; Ministerio de Economía y Competitividad(ES)(MDM-2017-0714).<br>EB, Canadian Network for Research and Innovation in Machining Technology,Natural Sciences and EngineeringResearch Council of Canada<br>RK, European Research Council (ERC) under the European Union’s Horizon 2020 research and innovation program (grant agreement No. 648693)

Powered by Editorial Manager® and ProduXion Manager® from Aries Systems Corporation

sure it is accurate.<br><br>**Unfunded studies**<br>Enter: *The author(s) received no specific funding for this work.*<br><br>**Funded studies**<br>Enter a statement with the following details:<br>• Initials of the authors who received each award<br>• Grant numbers awarded to each author<br>• The full name of each funder<br>• URL of each funder website<br>• Did the sponsors or funders play any role in the study design, data collection and analysis, decision to publish, or preparation of the manuscript?<br>• **NO** - Include this sentence at the end of your statement: *The funders had no role in study design, data collection and analysis, decision to publish, or preparation of the manuscript.*<br>• **YES** - Specify the role(s) played.<br><br>* typeset	
**Competing Interests**<br><br>Use the instructions below to enter a competing interest statement for this submission. On behalf of all authors, disclose any competing interests that could be perceived to bias this work—acknowledging all financial support and any other relevant financial or non-financial competing interests.<br><br>This statement **will appear in the published article** if the submission is accepted. Please make sure it is accurate. View published research articles from *PLOS ONE* for specific examples.	The authors have declared that no competing interests exist.

**NO authors have competing interests**<br><br>Enter: *The authors have declared that no competing interests exist.*<br><br>**Authors with competing interests**<br><br>Enter competing interest details beginning with this statement:<br><br>*I have read the journal's policy and the authors of this manuscript have the following competing interests: [insert competing interests here]*<br><br>* typeset	
**Ethics Statement**<br><br>Enter an ethics statement for this submission. This statement is required if the study involved:<br><br>• Human participants<br>• Human specimens or tissue<br>• Vertebrate animals or cephalopods<br>• Vertebrate embryos or tissues<br>• Field research<br><br>Write "N/A" if the submission does not require an ethics statement.<br><br>General guidance is provided below. Consult the submission guidelines for detailed instructions. **Make sure that all information entered here is included in the Methods section of the manuscript.**	The case study had the required university ethics approvals from McGill University.

Powered by Editorial Manager® and ProduXion Manager® from Aries Systems Corporation

**Format for specific study types**<br><br>**Human Subject Research (involving human participants and/or tissue)**<br>• Give the name of the institutional review board or ethics committee that approved the study<br>• Include the approval number and/or a statement indicating approval of this research<br>• Indicate the form of consent obtained (written/oral) or the reason that consent was not obtained (e.g. the data were analyzed anonymously)<br><br>**Animal Research (involving vertebrate animals, embryos or tissues)**<br>• Provide the name of the Institutional Animal Care and Use Committee (IACUC) or other relevant ethics board that reviewed the study protocol, and indicate whether they approved this research or granted a formal waiver of ethical approval<br>• Include an approval number if one was obtained<br>• If the study involved *non-human primates*, add *additional details* about animal welfare and steps taken to ameliorate suffering<br>• If anesthesia, euthanasia, or any kind of animal sacrifice is part of the study, include briefly which substances and/or methods were applied<br><br>**Field Research**<br><br>Include the following details if this study involves the collection of plant, animal, or other materials from a natural setting:<br>• Field permit number<br>• Name of the institution or relevant body that granted permission	
**Data Availability**<br><br>Authors are required to make all data underlying the findings described fully available, without restriction, and from the time of publication. PLOS allows rare exceptions to address legal and ethical concerns. See the PLOS Data Policy and FAQ for detailed information.	Yes - all data are fully available without restriction

A Data Availability Statement describing where the data can be found is required at submission. Your answers to this question constitute the Data Availability Statement and **will be published in the article**, if accepted.<br><br>**Important:** Stating 'data available on request from the author' is not sufficient. If your data are only available upon request, select 'No' for the first question and explain your exceptional situation in the text box.<br><br>Do the authors confirm that all data underlying the findings described in their manuscript are fully available without restriction?	
**Describe where the data may be found in full sentences. If you are copying our sample text, replace any instances of XXX with the appropriate details.**<br><br>• If the data are **held or will be held in a public repository**, include URLs, accession numbers or DOIs. If this information will only be available after acceptance, indicate this by ticking the box below. For example: *All XXX files are available from the XXX database (accession number(s) XXX, XXX.).*<br>• If the data are all contained **within the manuscript and/or Supporting Information files**, enter the following: *All relevant data are within the manuscript and its Supporting Information files.*<br>• If neither of these applies but you are able to provide **details of access elsewhere**, with or without limitations, please do so. For example:<br><br>*Data cannot be shared publicly because of [XXX]. Data are available from the XXX Institutional Data Access / Ethics Committee (contact via XXX) for researchers who meet the criteria for access to confidential data.*<br><br>*The data underlying the results presented in the study are available from (include the name of the third party*	This information will only be available after acceptance, indicate this by ticking the box below

and contact information or URL).<br>• This text is appropriate if the data are owned by a third party and authors do not have permission to share the data.<br><br>* typeset	
Additional data availability information:	Tick here if your circumstances are not covered by the questions above and you need the journal's help to make your data available.

Powered by Editorial Manager® and ProduXion Manager® from Aries Systems Corporation

Manuscript Click here to access/download;Manuscript;Manuscript.docx

Full name(s), professional affiliations and e-mail addresses of all author(s):

Amaia Albizua

Basque Centre for Climate Change (BC3), Parque Científico de UPV/EHU, Leioa, Spain

McGill University Macdonald-Stewart Building (Room 2-062), 21,111 Lakeshore Road, Ste. Anne de Bellevue, Québec, H9X 3V9, CANADA

amaiabc3@gmail.com

Elena M. Bennett

McGill University Macdonald-Stewart Building (Room 2-062), 21,111 Lakeshore Road, Ste. Anne de Bellevue, Québec, H9X 3V9, CANADA

elena.bennett@mcgill.ca

Guillaume Larocque

McGill University, Stewart Biology, McGill University, 1205 Avenue du Docteur-Penfield S3/18, Montréal, QC H3A 1B1, Canadá

guillaume.larocque@mcgill.ca

Robert W Krause

Institute for Analytical Sociology, Linköping University. Linköping University, The Institute for Analytical Sociology, 601 74 Norrköping, Sweden

robert.krause@liu.se

What affects farmers' advice networks: implications for agrarian sustainability

A. Albizua, E. Bennett, G. Larocque, and R. Krause

Abstract

Agriculture intensification, and its ecological and social effects, is pushing researchers to seek a rural transformation that supports biodiversity and agrarian ecosystems' functions. Social networks researchers have established that networks influence and are influenced by individuals' thoughts, values, and behaviors. However, there is incomplete knowledge about the factors that influence farmers' connections to seek advice about land management, and few studies addressing farmers' perceptions about their influence on agrarian ecosystems. We use interviews and social network data in a farming community undergoing accelerated agricultural intensification in Navarre, Spain to explore 1) farmers' perceptions regarding the effects of their management on the agrarian ecosystem and 2) the factors driving farmers' land management. We found that intensive farmers acknowledge their practices' negative effects on the agrarian ecosystem, and small-scale farmers recognize their contribution to traditions, traditional knowledge, landscape, and their capacity to regulate the climate. However, farmers negatively affecting the ecosystems control the information flow in our studied rural network whereas farmers aware of their contribution to climate regulation ask more for advice from other groups of farmers while they are not frequently asked for advice. Those results inform rural policies about how and why intensive practices dominate over sustainable practices and uncover that incentivizing small-scale farmers' participation in the advice network could decrease the current obstacles for farmers' engagement with sustainable farming practices.

Keywords: social network structure, social network analysis, agriculture intensification, sustainability

1. Introduction

Farmers are intensifying their agrarian practices worldwide (1). There are more industry-oriented farms, characterized by large-scale monocultures with high use of pesticides and fertilizers and, often, irrigation inputs. At a global level, this intensification is driven by a desire for higher yields to feed a growing population whose changes in diet are influenced by rapid economic growth in much of the developing world (2). Energy demand pressures also drive large-scale buyers and transformers to allocate growing amounts of food crops to provide biofuels, as a solution to high fuel prices and energy insecurity (3). However, agricultural intensification is a major driver of biodiversity loss, which leads to a landscape simplification and ultimately avoids the maintenance of communities at the landscape scale (4). There are other secondary effects such as a decrease in pollination services (5), pest spread (6), increased land erosion (7), and water pollution (8). Moreover, intensification of agrarian systems also alters institutions and social relations (9), displacing agriculture alternatives such as small-scale and organic farming.

Most of those environmental problems are not directly addressable by either central governments or individuals because they emerge from the interdependent actions of many decision-makers (10). If farmers aim to improve their surrounding underground water, they can opt to use organic fertilizers and reduce pesticide spreads. However, if the rest of the neighbor farmers do not follow suit, the impacts might be minimal at the landscape level. Social networks enable interactions across scales that can support spreading (un)sustainable land management practices (11). This is, farmers, embedded in social networks, exchange information and resources with other farmers and institutions, who simultaneously interact with other institutions at different scales (local, regional, national) (12). Those multi-scale interactions allow co-operative actions and dissemination of the “know-how” between rural community members, ultimately contributing to scaling up and out the dominant management practices shared in the network (11).

There are some studies about farmers' perceptions and values regarding Nature Contributions to People (hereafter NCP) (13–15) or about the factors influencing farmers' networks to understand how peers influence each other in their land management activities and ideas (16–18). However, farmers' self-perception about their co-production of bundles of NCP and what affects farmers' interactions when they make land management decisions (i.e. the way such a rural network structure emerges) deserve further research (19,20).

Our first objective is to explore farmers' views on their role in co-producing positive and negative effects of their farming practices. Farmers' awareness of their potential to influence multiple services is crucial since it represents the first step for action. We hypothesize that farmers' perceptions about NCP are aligned with their land management practices and, thus, with the potential to co-produce certain NCP over others. An example could be that farmers developing intensive land management are aware of obtaining higher yields but they may also aware of deteriorating biodiversity or traditional knowledge and landscape aesthetics. Our second objective is to examine to what extent structurally driven relationship formation (e.g., transitivity, understood as that advisor of advisors is also sought for advice), and attributed driven factors (e.g., being of similar age), influence the connections among farmers regarding land management. Our second hypothesis is that farmers sharing similar land management practices and NCP co-production perceptions are significant factors for the network ties' creation. We use data from a farming community in a village in the region of Navarre, Spain. The agricultural practices in this village are undergoing intensification, which affects farmers' informal networks as well as their awareness of the impact of their management on NCP (9,15,21).

We believe this information is useful in interpreting variation in farmers' engagement with the agrarian ecosystem (22) and, ultimately understand the ecological consequences of their land management decisions (23). This is especially relevant in a context where there are increasing

resources devoted to rural sustainability, (e.g., Communitarian Agrarian Policy designed schemes), but farmers' engagement with sustainability is still very low (24,25).

2. Conceptual Framework

Nature's contributions to people

Agrarian ecosystems offer many contributions beyond the production of food (16). Farmers' land management influences the regulation of NCP (e.g. pollination or soil and groundwater quality) as well as non-material NCP (e.g. landscape aesthetics or traditional knowledge maintenance). The farming strategy uptake largely depends on farmers' capacity, this is their access to knowledge and resources (e.g., technical, such as machineries like tractors or irrigation infrastructure, and financial such as subsidies that aid to access and maintain the technology) determining also their identity, understood as what is meaningful and important to them.

In Europe, irreplaceable cultural landscapes associated with traditional agricultural practices are disappearing because of agricultural intensification (26,27), which may also change farmers' perceptions about their responsibility for co-producing a variety of NCP (28). Here we adopt the IPBES NCP framework (16,29) to incorporate NCP research into decision-making for the sustainable use of natural resources (30).

Social network structure and its drivers

Social network analysis (SNA) focuses on the structure of ties among a set of actors. In our case, the actors are farmers and the ties are land management related knowledge exchanges. Three comprehensive drivers influence the formation of social ties (31): (1) network structure; (2) nodal attributes, and (3) external contextual factors. For network structure, we refer to one in which two or more farmers share rural advice. These farmers can get into a partnership for land management, or not. Nodal attributes refer to the farmers' characteristics, such as their perceptions, age, gender, etc. When the social tie formation depends on attributes of the nodes

and how these attributes interact with the network structure (e.g., differences in network activity) and with the attributes of other nodes (e.g., homophily) we talk about nodal attribute drivers (31,32). Finally, external contextual factors are factors that do not depend on farmers, such as the lithology of the land they cultivate, or the distance between plots. These drivers help understand how patterns of ties, and thus social networks, emerge.

While network and nodal attribute drivers are endogenous to the network, exogenous contextual factors can also play a major role in the network formation. The cultural, social, geographic, and ecological environments of the network can be crucial for the creation and maintenance of social ties. The analysis we propose in this article focuses on understanding the role and interplay of the different internal and external drivers.

3. Materials and methods

3.1. Study area

We studied a farming community from a village of the Navarre region, in Spain. The name of the village will remain anonymous to protect the identity of the participants in the study. This village underwent agricultural intensification through the adoption of large-scale irrigation that began in 2006 and it is still undergoing. This has affected farmers' land management and perceptions about their practices' impacts.

Fig 1. Location of Navarre province, Spain.

The adoption of large-scale irrigation has led to several changes in land management and farmers association and behavior (12). There has been an increase in the size of the cropped land per farmer; farmers have introduced new crops such as corn (Zea mays), forage, or biofuels

plantations (33). Moreover, farmers now employ pressure sprinklers and use higher doses of mineral fertilizers and pesticides.

Agricultural intensification in this region of Navarre is leading to higher nitrate pollution of land and water, both in rivers and underground water (34), among other negative environmental impacts (35). Likewise, alternative-farming practices such as small-scale and organic farming are being displaced (9).

Land and labor allocation dynamics have also changed in the region. In this regard, local cooperatives, after the large-scale irrigation project installation, and because of land concentration to cultivate larger plots, coordinate farmers and landholders' land and labor exchanges (12). Many old farmers not willing to invest in the new technology have abandoned their farming activity and they often rent their lands to other farmers for cultivation (9). Normally, land management is allocated to younger and capable farmers (in terms of adapted technology). Consequently, many landholders leave their land to such cooperatives that distribute the land among other active farmers for cultivation. Moreover, new kinds of cooperatives, such as those for ownership of machinery, have been created to share new machinery between small groups of farmers.

3.2. Data and methods

Sampling for the social network analysis

We used a list of all landowners and farmers affected by the Navarre government’s large-scale irrigation project, provided to us by the irrigation community, alongside insight from key local informants (e.g. agrarian cooperative workers) to identify members of the community. We used snowball sampling until no additional farmers or landholders’ names were generated.

We got a response rate of 77% through the combination of the list and the snowball sampling (81 farmers and landowners out of the 106 people approached). The diversity of farmers was

purposely searched (see (15)). The surveyed farmers nominated 80 additional people (i.e. farmers and other rural community members such as rural advisors, cooperative workers, and sellers) leading to a total network of 161 people. However, only the originally approached farmers that responded to our survey (N=81) are included in the Exponential Random Graph Model (ERGM) (below explained). Only these respondents provided information about their NCP perceptions and management behavior, which are crucial factors of our analysis.

We collected data with in-person semi-structured interviews with farmers and landholders during June-August 2017. Through the interviews, a name generator with a free recall was used (31). We asked farmers to mention up to five people (36) who they consider influencers in their land management decision-making. We later asked about their perceptions regarding which kind of NCP they enhanced through their land management and farming choices. We asked respondents both with whom they share knowledge and from whom they receive knowledge. We asked openly about the knowledge exchange, which helped to make a list of possible topics they talked about and which was later used when farmers seemed to have difficulties remembering (**Figure 2**).

Fig 2. The sampling of participants for social network analysis.

Semi-structured interviews

We performed thirty-two additional interviews with organizations related to the local agrarian sector. The surveyed farmers had mentioned 27 of the organizations interviewed. To understand how other organizations can influence farmers' land management decision-making (see **Figure 3** and supplementary material), five other organizations were selected based on a literature review and the experience of the lead author who has been researching in the area since 2013. This

additional information is considered relevant to better understand the context and to triangulate the information so that we can make a better interpretation of the results.

Fig 3. The sampling of participants for the interviews.

Hierarchical cluster analysis to understand rural community composition

We performed two hierarchical cluster analysis (HCA) to classify farmers and landholders into two sets of groups, according to 1) their land management decision-making (Management Cluster- MC) and, 2) their perceptions regarding which NC they enhance with their land management (Nature Contribution to People Cluster - NCPC). Grouping farmers concerning their farming practices allow identifying, to a limited extent, their engagement with the ecosystem.

Nature contribution to people co-production awareness differences among farmers' groups

We asked farmers about 14 different types of positive and negative contributions they may enhance through their management. These included instrumental values such as the provision of food, habitat, pest control, or climate regulation; as well as relational values such as physical and mental health and cultural identity. Those NCP were selected based on previous work (15) developed in the region in which socio-cultural values over NCP were identified.

First, we explored if there were significant differences in some management variables among the three groups of farmers with differentiated NCP co-production awareness. Second, we examined the NCP co-production awareness among the three groups of farmers with different land management strategies using a Kruskal-Wallis test (37–39). A False Discovery Rate (FDR) control approach was used to counteract the problem of multiple comparisons (40).

Selection of drivers to understand social tie formation before their inclusion in the Exponential random graph model (ERGM)

We used an Exponential Random Graph Model (ERGM) - i.e. a statistical model for analyzing the structural features of an observed network where its parameters depend on the frequency of occurrence of substructures in the network (41). This is, a statistical model should consider the set of all possible alternative networks weighted on their similarity to an observed network to support statistical inference on the processes influencing the formation of network structure. The ERGM gives each of the examined indicators an estimated parameter and a standard error. A positive parameter means that the respective subgraph (e.g., number of reciprocated ties) is occurring more often than expected by chance, given the rest of the model, while a negative parameter means that the respective subgraph is occurring less often than expected by chance, given the rest of the model. For a detailed introduction into ERGMs see (42).

To understand the role of farmers' network structures on land management decision-making, we took the mixed exploratory approach and hypothesis-driven approaches testing the following structurally driven tie formation factors: reciprocity, transitivity, network activity, and network popularity (32,43). We removed the last two, network activity, and network popularity because they were not significant.

Transitivity, in this context, means that advisors of advisors are also sought for advice. Network activity and popularity refer to the number of outgoing ties that are the number of other people asked for advice from a farmer, and incoming ties, or the number of people seeking advice from the farmer. These indicators show the presence of leaders, who are consulted more frequently due to their position, experience, and knowledge. Moreover, (44) showed that leaders can bring people together and may influence others' values and perspectives (17,18,45).

We also included attribute driven factors influenced by participants' characteristics. Homophily is the tendency for actors to seek information from those who share the same characteristics.

Precisely, we tested homophily in social variables such as age, NCP perceptions, and land management, all extracted from the surveys, to see if those factors influenced the network configuration. Sharing information with others that practice similar farming strategies is recognized as a likely practice (46).

Regarding biophysical contextual factors, we included geographic proximity in the model, which has been shown (e.g., (43)) to be a major contributor to network formation. The presence of a plot within 1 km was selected as the distance metric, with the expectation that farmers located nearby are more likely to be asked for advice than distant farmers. See Table 3 in the Supplementary Material for a more detailed justification of why other geographic and ecological environment-related variables could not finally be included in the model.

Exponential Random Graph Model (ERGM)

ERGMs permit testing all of these variables simultaneously and predicting edge formation (42), therefore informing about how land management related rural networks are created. We fit the ERGMs using the ergm (v2018.10, Handcock and Gile, 2010; Handcock et al., 2014) software package for the R (R version 3.6.3) programming language (R Team et al., 2020). We constrained the simulations to a maximum of 5 nominees.

The ERGM was used to test whether land management related advice relationships are associated with a similarity of farming types, social characteristics, and geographical proximity. Sometimes, especially when the number of configurations included is high or the models are not well specified, ERGMs do not converge (31). To deal with this we started with a simple model and added more configurations incrementally if there were reasons to believe they were important (see Table 2 in the Supplementary Material regarding our reasoning to include environmental variables). After obtaining a structural baseline model, we first tested each new set of parameters, added to the simple, smaller model, and then later tested a final model with all the previously significant parameters. This way we can explore the data in several directions and

preselect relevant effects while reducing the number of false positives by testing all relevant effects jointly in a final model.

Missing data

Generally, farmers managing land answered about their co-production of NCP awareness. However, some of the landholders who did not manage land anymore did not answer this question, and, for this reason, we took a subsample of 55 people regarding farmers' perception of NCP co-production.

ERGMs can be reliably estimated under missing data (assuming the data is missing at random), as long as the missing data is only on the network ties (47). Missing covariates, however, cannot easily be handled within the ERGM framework because the model is only generative regarding network links and treats all covariates as exogenous (this means that missing covariate values cannot be internally imputed during the estimation). We thus base our findings for the ERGM only on a subsample of 80 respondents on which we have nearly complete information. One farmer was the only one who used purely organic farming techniques and asked no-one else for advice, nor did anyone ask this farmer for advice. This farmer thus represented a very special case and was excluded. It has been shown that ERGMs still provide reliable results with 20-30% missing data (48,49).

4. Results

Farmers perceptions about their role in NCP co-production

We found different levels of awareness among farmers regarding how they can affect (positively or negatively), through their land management, NCP in agrarian ecosystems.

Not surprisingly, the provision of food was the most highly cited contribution, followed by some cultural contributions such as farmers' recreation and their influence on landscape configuration. They also mentioned their effect on creating an adequate habitat to enhance biodiversity and

their role in water regulation. In contrast, their effect on land pollution and climate regulation was the least cited. See Figure 4 to identify which NCP were perceived as being highly influenced by farmers' farming in order of their attached importance.

Fig 4. Percentage of farmers aware of their NCP perceived as co-produced through their farming

Differentiated groups of farmers

We identified two distinct groups of farmers through two different Hierarchical Cluster Analysis (HCA). The result of the first HCA grouped farmers according to their land management (Management Cluster- MC) into three groups: intensive farmers (N=45), small-scale farmers (N=21), and landowners (N=15). The second HCA was based on their perception of their role in the co-production of NCP (Nature Contribution to People Cluster - NCPC) and it revealed three different groups: farmers composed of those conscious of their negative impacts or disservices (N=19); farmers aware of their contribution to climate regulation and landscape aesthetics (N=42); and those aware of their role to enhance the habitat conditions, traditional knowledge, traditions maintenance, and regulation of pests (N=20). The interviews showed that this last group probably kept traditional ways of farming that were especially adequate for pest regulation. Most likely, these practices had been learned from their parents or neighbors and they considered that keeping such farming practices contributed to keeping traditional knowledge, rural traditions, and educating other generations. (See Table 1 for more detail on the characteristic of each group).

Table 1. Network nodes characteristics in the two Hierarchical Cluster Analysis (Based on 80 community participants)

**HCA1 based on management**	**HCA2 based on NCP co-production awareness**
Intensive farmers (N=48) hold large areas with agro-industry oriented crops (maize, grass, biofuels, cereals), sprinkling irrigation, mixed fertilization They make all decisions about farming and investments. They normally contract labor.	*Conscious of their negative impacts* (N=19) They realize their practices enhance soil erosion, land pollution, and habitat degradation.
Small-scale farmers (N=44) hold plots between 0-5 hectares of “other” crops (vegetables, fruit trees such as olive or almond trees). Hardly any sprinkling irrigation, not main commercial crops (maize, vineyards, and biofuels).<br>Mixed variety of fertilizers. Normally small-scale farmers do not rely only on agriculture, but have other sources of income or are retired farmers.	*Climate regulation and landscape advocates* (N=42).<br>These farmers consider they helped on climate change mitigation through the store of carbon underground and above ground in more perennial crops such as wooden crops. Likewise, they are aware that their farming practices, types of crops, and rotations contribute to improving landscape aesthetics.
Landholders (N=29) are not directly associated with farming and they do not make decisions on it either on technology investments. They are normally retired farmers who own many small surfaces of	*Traditionalists and habitat supporters*<br>Those farmers enhance regulating and cultural contributions (N=20) Aware of their role to enhance the habitat conditions,

arable land in the village and rent those lands to other farmers to labor them.	traditional knowledge, traditions maintenance, and regulation of plagues.

Differences among farmer groups regarding management and NCP co-production awareness

We did not find any significant difference (after adjusting for multiple comparisons) among the farmers performing different kinds of land management. However, we did find some tendencies regarding some management choices, such as the type of irrigation and fertilizer use, some crop selection, and, to a lower extent, the surface labored, influenced significantly the NCP co-production perceptions (see Figure 5 where significance refers to the Kruskal-Wallis test before FDR being applied). Those using dropping irrigation and who normally cultivated “other” crops encompassing wooden fruit trees and vegetables were normally aware of their contribution to climate regulation, landscape aesthetics, traditions, and habitat. In contrast, those cultivating cereals (normally in large plots) and using mineral fertilizers were aware of their management’s negative impacts on the agrarian ecosystem. Interviews showed that those aware of their negatives effects particularly thought they were eroding and polluting the land. They also showed a consciousness about their lack of cultural benefits enhancement.

Fig 5. Land management differences among the groups of farmers with different NCP co-production awareness

‘*’ $p < .05$; ‘**’ $p < .01$ Kruskal Wallis test, without adjustment for multiple comparisons.

When assessing how different types of farmers (clusters based on their management) perceived their NCP co-production, we neither found any significant difference among the group of farmers but we did find some tendencies: small-scale farmers (who cultivate small plots, grow

vegetables and fruit trees with drip irrigation systems and mixed fertilizers) had a higher awareness of their role in enhancing traditional knowledge, traditions, and landscape aesthetics (see Figure 6 where significance refers to the Kruskal-Wallis test before FDR being applied). Likewise, (but to a lower extent) they also thought they contributed to climate regulation. For more details on those differences see Tables 2 and 3 in the Supplementary Material.

Fig. 6. NCP co-production awareness differences among the groups of farmers with distinct land management strategies.

Significance codes '*' $p < .05$; '**' $p < .01$, without adjustment for multiple comparisons.

ERGM results

The results of the ERGM (Table 2) indicate that advice seeking is strongly reciprocal; that is, farmers are likely to ask advice from farmers that also ask them for advice in return, and highly transitive, as can be seen in the positive gwesp term. Neither gw-in- nor outdegree are significant, that is, there are no significant hubs in the network.

Farmers sharing the same crops are more likely to seek advice from each other while farming land close to each other or the age-difference does not significantly influence the advice-seeking behavior.

The results for the Management cluster reveal that old landholders (MC3) are more likely to be asked for advice (reference category MC1), and small-scale farmers (MC2) and old landholders (MC3) are less likely to ask for advice. For the NCPC awareness clusters, we find that farmers who think they contribute to climate regulation and landscape aesthetics (NCPC2) are less likely

to be asked for advice, and more likely to ask others for advice. There seems to be no preference to ask farmers within the same NCPC or management behavior for advice.

Additionally, a more detailed analysis of the advice-seeking behavior modeling the connectivity between the management clusters revealed that small-scale farmers (MC2) do not often ask each other for advice, nor do they often ask old landholders (MC3) for advice. Likewise, old landholders (MC3) do not seek advice from small-scale farmers (MC2). These results indicate that large-scale intensive farmers (MC1) are crucial for the information flow throughout the community.

Table 2. ERGM results for farmers advice-seeking presenting the estimated value in the model for each of the parameters considered (Estimate), their standard error (SE) and the p-value (p)

Parameter	Estimate	SE	*p*
**Edges (ties)**	**-5.594**	**0.673**	**<.001**
**Mutual (reciprocity)**	**7.472**	**0.638**	**<.001**
**gwesp(□ = 0.3) (transitivity)**	**0.457**	**0.121**	**<.001**
**gw-indegree(□ = 0.9) (popularity)**	**0.098**	**0.505**	**.845**
**gw-outdegree(□ = 0.1)**	**-0.770**	**0.544**	**.157**
absolute age difference (homophily age)	-0.011	0.006	.083
**shared crops** (homophily crops)	**0.207**	**0.076**	**.006**
distance (1km)	-0.147	0.143	.304
indegree MC2[a] (homophily management)	0.559	0.693	.420
**indegree MC3[a] (homophily management)**	**1.363**	**0.672**	**.043**
**outdegree MC2[a] (homophily management)**	**-2.463**	**0.753**	**<.001**
**outdegree MC3[a] (homophily management)**	**-2.637**	**0.671**	**<.001**
same MC (homophily management)	-0.167	0.488	.732
**indegree NCPC2[b] (homophily perception)**	**-0.897**	**0.366**	**.014**

indegree NCPC3[b] (homophily perception)	-0.114	0.411	.781
**outdegree NCPC2[b] (homophily perception)**	**0.942**	**0.351**	**.007**
outdegree NCPC3[b] (homophily perception)	0.063	0.425	.883
same NCPC (homophily perception)	-0.190	0.140	.175

Note. AIC: 784.5 BIC: 906.

[a]Reference category: MC1

[b]Reference category: NCP1

Complementary to ERGM results, Figure 5 reveals that large-scale intensive farmers occupy central positions and are very active in advice sharing, both giving and receiving advice about land management. In contrast, most small-scale farmers are in the periphery and often isolated, not being asked or asking for advice to others. Such small-scale farmers are traditionalists and are aware of their role to support the agrarian ecosystem habitat, as well as those aware of their capacity to regulate climate and contribute to landscape configuration (Figures 3, 4 and 5). Therefore, we can deduce that farmers aware of their regulating and cultural NCP do not play an important role in this network. However, intensive farmers control the information flow, being able to spread unsustainable practices in this community. Figure 5 allows us to visualize how farmers performing intensive practices dominate over sustainable practices, even when such intensive farmers recognize their impacts on the agrarian ecosystem.

Fig 7. The advice network of the farming community in the village of Navarre in 2017.

5. Discussion and conclusions

We found that most farmers were aware of their co-production of bundles of NCP through their land management. Moreover, we discovered that farmers' perceptions regarding how they contributed to NCP were aligned with their land management decision-making. Intensive farmers cultivating large plots (>100 Ha) of irrigated cash crops using mineral fertilizers tended to be aware of the negative impacts of their land management, as well as their lack of cultural contribution. On the other hand, small-scale farmers acknowledged their non-material NCP related to climate change regulation, traditional knowledge, traditions, and landscape configuration.

We also discovered that farmers' perceptions about NCP co-production and their land management were significantly important in rural network creation. Farmers sharing similar land management practices and NCP co-production perceptions were significant factors for the network ties' creation. Intensive farmers were most active and occupied central positions in the exchange of farming advice. In contrast, small-scale farmers were often isolated (Figure 7), not sharing their farming knowledge with the rest of the farmers.

Therefore, despite both groups of farmers being aware of their negative and positive contributions, respectively, those conscious of their management degradation effects shared farming advice whereas those aware of their contribution to climate and pest regulation as well as other cultural benefits kept their land management knowledge for their own.

The mechanisms by which social networks could be used to promote activities for adaptation-mitigation (11) are also applicable to the spread of (un)sustainable farming practices. This study probes the roles and responsibilities of intensive vs. small-scale farmers when choosing different co-production pathways (50). Moreover, the cognitive and management dimensions linked to the creation of a rural advice network are critical to understanding how dominant farming practices (intensive practices in this case), at a local scale, can be spread at a landscape scale through the combination of networks between farmers, farmers and rural institutions, and between institutions (11,12).

Several factors can help to understand the social mechanisms behind our results. It seems that education about the consequences of certain farming practices is not enough to promote sustainable farming practices. Intensive farmers already know about their land management consequences but keep performing management that undermines the agrarian ecosystem. This result aligns with what (24) found: it was not a lack of knowledge about the required agricultural changes that prevented EU agricultural subsidies from achieving their environmental objectives.

We argue that the main reason is not a short-term vision of influential farmers but rather a more systematic and structural cause. The integration of rural economies into global commodity markets makes intensive farmers dependent on high yields that can only be obtained through intensive practices (Kay, 2002; Tilman et al., 2011; Cramb, 2011).

Certainly, there are still small-scale farmers performing a different (and probably more sustainable) land management, and who are aware of their immaterial but still important NCP. However, interviews showed that those farmers cannot generally live on their farming and they cultivate their land as a complement to other jobs or as a hobby (9). The introduction of large-scale irrigation seems to be exposing those small-scale farmers to unexpected losses in local knowledge and the substitution of crops not suiting carbon store capacity, like fruit trees. Further research is needed regarding how those types of farms are becoming invisible for policies and markets, making at the same time very difficult their interests be supported, and consequently, their intangible contributions, such as traditional knowledge, can be lost over time (51).

Regarding the endogenous network processes, - i.e. those belonging to their networks, such as network and nodal attributes-, reciprocity and transitive closure play an important role. These are typical findings for social networks (Siciliano, 2015; Thomas and Caillon, 2016). Giving advice and providing help costs resources and is often repaid by advice and help in return. Further, in our measurement, we aggregated several different advice-seeking topics. Given that some individuals are more knowledgeable or experienced than others in certain topics, it is natural that advice-givers also exchange advice between themselves, leading to transitive closure.

Aligned with our expectations, farming practices, and farmers' perceptions about NCP co-production, did significantly influence the network formation. There was no preference to ask for advice from farmers who have the same NCP perceptions or land management practices. Consequently, most intensive farmers controlled the flow of information since small-scale

farmers and landholders did not normally ask each other for advice. Intensive farmers sought, however, more advice from landowners than they did from each other or small-scale farmers. This finding may be related to the fact that intensive farmers normally cultivated landholders' plots and, therefore, asked them about their land management preferences. Moreover, those farmers aware of their contribution to climate change regulation and landscape configuration were less likely to be asked for advice but more likely to ask others for land management advice. This pattern suggests that in our data, sustainable farming practices are less likely to spread through the network.

In contrast, exogenous factors, such as proximity to other farmers, were not a driving factor in forming network structure (as happened Isaac and Dawoe, 2011 in their case study about agroforestry advice networks in Ghana). Houses proximity may be more important than the proximity of the cultivated plots or easily available modern technology (e.g. mobile telephones) reduces the importance of shorter distances.

Our study has some limitations. It can be difficult to make generalizations with a case study of a small community. However, we find important contextual similarities with other rural communities in Western Europe (Labarthe and Laurent, 2013) and worldwide (Kay, 2002). Additionally, we did not have longitudinal data on the networks and attributes. This limits our ability to assess how advice networks in rural communities influence farmers' land management decision making and their perceptions about it, as well as, simultaneously, how those dynamic variables affect the formation of rural networks (see, e.g., Steglich, Snijders, and Pearson, 2010, for the problem of how to disentangle network selection and network influence processes).

Despite those caveats, this rural network analysis is relevant for understanding the network configuration of rural farming communities and for rural policy development since it permits understanding interactions at a local level. Being aware of the control executed by intensive farmers informs about the less likely spread of sustainable farming practices given that intensive

farmers also interact more often with cross-scale rural institutions (12). Considering that those farmers' perceptions and their management practices are significant factors for the creation of the advice network structure, we should encourage farmers like those aware of their contribution to climate regulation to take a more active role within their networks. Incentivizing small-scale farmers' practices diffusion through their higher participation in advice-giving structures (e.g. within the local cooperatives), as well as withdrawing some power from intensive farmers could be one way of changing this situation.

Our case shows that intensive farmers might influence the rest of the community to adopt their farming strategies. Moreover, explicit acknowledgment of how networks operate concerning farming advice, thus, has the potential to be a critical ingredient when designing and implementing rural policies seeking for sustainable agriculture.

References

Albizua, A., Corbera, E., Pascual, U., 2019a. Farmers' vulnerability to global change in Navarre, Spain: large-scale irrigation as maladaptation. Regional environmental change 19, 1147–1158. https://10.1007/s10113-019-01462-2

Albizua, A., Pascual, U., Corbera, E., 2019b. Large-scale irrigation impacts socio-cultural values: an example from rural Navarre, Spain. Ecological economics 159, 354–361. https://doi.org/10.1016/j.ecolecon.2018.12.017

Albizua, A., Zaga-Mendez, A., 2020. Changes in institutional and social–ecological system robustness due to the adoption of large-scale irrigation technology in Navarre (Spain). Environmental Policy and Governance. https://doi.org/10.1002/eet.1882

Albizua, A., Bennett, E., Pascual, U., Larocque, G., 2020. The role of the social network structure on the spread of intensive agriculture: An example from Navarre, Spain. Regional Environmental Change. https://10.1007/s10113-020-01676-9

Alexander, S., Bodin, Ö., Barnes, M., 2018. Untangling the drivers of community cohesion in small-scale fisheries [WWW Document]. URL https://www.thecommonsjournal.org/articles/10.18352/ijc.843/ (accessed 4.26.18).

Barnaud, C., Corbera, E., Muradian, R., Salliou, N., Sirami, C., Vialatte, A., Choisis, J.-P., Dendoncker, N., Mathevet, R., Moreau, C., 2018. Ecosystem services, social interdependencies, and collective action: a conceptual framework. Ecology and Society 23. https://10.5751/ES-09848-230115

Barnosky, A.D., Matzke, N., Tomiya, S., Wogan, G.O., Swartz, B., Quental, T.B., Marshall, C., McGuire, J.L., Lindsey, E.L., Maguire, K.C., 2011. Has the Earth's sixth mass extinction already arrived? Nature 471, 51–57. https://doi.org/10.1038/nature09678

Bennett, E.M., Cramer, W., Begossi, A., Cundill, G., Díaz, S., Egoh, B.N., Geijzendorffer, I.R., Krug, C.B., Lavorel, S., Lazos, E., Lebel, L., Martín-López, B., Meyfroidt, P., Mooney, H.A., Nel, J.L., Pascual, U., Payet, K., Harguindeguy, N.P., Peterson, G.D., Prieur-Richard, A.-H., Reyers, B., Roebeling, P., Seppelt, R., Solan, M., Tschakert, P., Tscharntke, T., Turner, B., Verburg, P.H., Viglizzo, E.F., White, P.C., Woodward, G., 2015. Linking biodiversity, ecosystem services, and human well-being: three challenges for designing research for sustainability. Current Opinion in Environmental Sustainability, Open Issue 14, 76–85. https://doi.org/10.1016/j.cosust.2015.03.007

Berka, C., Schreier, H., Hall, K., 2001. Linking water quality with agricultural intensification in a rural watershed. Water, Air, and Soil Pollution 127, 389–401. https://10.1023/A:1005233005364

Bodin, Ö., Tengö, M., 2012. Disentangling intangible social–ecological systems. Global Environmental Change 22, 430–439. https://10.1016/j.gloenvcha.2012.01.005

Boserup, E., 2014. The conditions of agricultural growth: The economics of agrarian change under population pressure. Routledge.

Brondizio, E.S., Ostrom, E., Young, O.R., 2009. Connectivity and the governance of multilevel social-ecological systems: The role of social capital. Annual review of environment and resources 34, 253–278. https://doi.org/10.1146/annurev.environ.020708.100707

Brown, S.R., 2006. A match made in heaven: A marginalized methodology for studying the marginalized. Quality and Quantity 40, 361–382. https://doi.org/10.1007/s11135-005-8828-2

Bruinsma, J., 2017. World agriculture: towards 2015/2030: an FAO study. Routledge.

Colloff, M.J., Martin-Lopez, B., Lavorel, S., Locatelli, B., Gorddard, R., Longaretti, P.-Y., Walters, G., van Kerkhoff, L., Wyborn, C., Coreau, A., 2017. An integrative research framework for enabling transformative adaptation. Environmental Science & Policy 68, 87–96. https://doi.org/10.1016/j.envsci.2016.11.007

Cramb, R.A., 2011. Agrarian transitions in Sarawak: Intensification and expansion reconsidered. Borneo transformed: Agricultural expansion on the Southeast Asian frontier 44–93.

Crona, B., Gelcich, S., Bodin, Ö., 2017. The importance of interplay between leadership and social capital in shaping outcomes of rights-based fisheries governance. World Development 91, 70–83. https://doi.org/10.1016/j.worlddev.2016.10.006

Díaz, S., Demissew, S., Carabias, J., Joly, C., Lonsdale, M., Ash, N., Larigauderie, A., Adhikari, J.R., Arico, S., Báldi, A., Bartuska, A., Baste, I.A., Bilgin, A., Brondizio, E., Chan, K.M., Figueroa, V.E., Duraiappah, A., Fischer, M., Hill, R., Koetz, T., Leadley, P., Lyver, P., Mace, G.M., Martin-Lopez, B., Okumura, M., Pacheco, D., Pascual, U., Pérez, E.S., Reyers, B., Roth, E., Saito, O., Scholes, R.J., Sharma, N., Tallis, H., Thaman, R., Watson, R., Yahara, T., Hamid, Z.A., Akosim, C., Al-Hafedh, Y., Allahverdiyev, R., Amankwah, E., Asah, S.T., Asfaw, Z., Bartus, G., Brooks, A.L., Caillaux, J., Dalle, G., Darnaedi, D., Driver, A., Erpul, G., Escobar-Eyzaguirre, P., Failler, P., Fouda, A.M.M., Fu, B., Gundimeda, H., Hashimoto, S., Homer, F., Lavorel, S., Lichtenstein, G., Mala, W.A.,

Mandivenyi, W., Matczak, P., Mbizvo, C., Mehrdadi, M., Metzger, J.P., Mikissa, J.B., Moller, H., Mooney, H.A., Mumby, P., Nagendra, H., Nesshover, C., Oteng-Yeboah, A.A., Pataki, G., Roué, M., Rubis, J., Schultz, M., Smith, P., Sumaila, R., Takeuchi, K., Thomas, S., Verma, M., Yeo-Chang, Y., Zlatanova, D., 2015. The IPBES Conceptual Framework — connecting nature and people. Current Opinion in Environmental Sustainability, Open Issue 14, 1–16. https://doi.org/10.1016/j.cosust.2014.11.002

Díaz, S., Pascual, U., Stenseke, M., Martín-López, B., Watson, R.T., Molnár, Z., Hill, R., Chan, K.M.A., Baste, I.A., Brauman, K.A., Polasky, S., Church, A., Lonsdale, M., Larigauderie, A., Leadley, P.W., Oudenhoven, A.P.E. van, Plaat, F. van der, Schröter, M., Lavorel, S., Aumeeruddy-Thomas, Y., Bukvareva, E., Davies, K., Demissew, S., Erpul, G., Failler, P., Guerra, C.A., Hewitt, C.L., Keune, H., Lindley, S., Shirayama, Y., 2018. Assessing nature's contributions to people. Science 359, 270–272. https://doi.org/10.1126/science.aap8826

Fischer, A., Eastwood, A., 2016. Coproduction of ecosystem services as human–nature interactions—An analytical framework. Land Use Policy 52, 41–50. https://doi.org/10.1016/j.landusepol.2015.12.004

Foley, J.A., DeFries, R., Asner, G.P., Barford, C., Bonan, G., Carpenter, S.R., Chapin, F.S., Coe, M.T., Daily, G.C., Gibbs, H.K., 2005. Global consequences of land use. Science 309, 570–574. https://10.1126/science.1111772

Frank, O. and Strauss, D. (1986). Markov graphs. Journal of the American Statistical Association, 81:832--842. DOI: 10.1080/01621459.1986.10478342

Gámez-Virués, S., Perović, D.J., Gossner, M.M., Börschig, C., Blüthgen, N., De Jong, H., Simons, N.K., Klein, A.-M., Krauss, J., Maier, G., 2015. Landscape simplification filters species traits and drives biotic homogenization. Nature communications 6, 1–8. https://doi.org/10.1038/ncomms9568

García-Llorente, M., Martín-López, B., Díaz, S., Montes, C., 2011. Can ecosystem properties be fully translated into service values? An economic valuation of aquatic plant services. Ecological Applications 21, 3083–3103. https://doi.org/10.1890/10-1744.1

Gile, K., Handcock, M.S., 2006. Model-based assessment of the impact of missing data on inference for networks. CSSS Working Paper No. 66, University of Washington, Seattle.

Groce, J.E., Farrelly, M.A., Jorgensen, B.S., Cook, C.N., 2019. Using social-network research to improve outcomes in natural resource management. Conservation biology 33, 53–65. https://10.1111/cobi.13127

Handcock, M.S., Gile, K.J., 2010. Modeling social networks from sampled data. The Annals of Applied Statistics 4, 5-25 https://10.1214/08-AOAS221

Handcock, M.S., Gile, K.J., Mar, C.M., 2014. Estimating hidden population size using respondent-driven sampling data. Electronic journal of statistics 8, 1491.

Iniesta-Arandia, I., García-Llorente, M., Aguilera, P.A., Montes, C., Martín-López, B., 2014. Socio-cultural valuation of ecosystem services: uncovering the links between values, drivers of change, and human well-being. Ecological Economics 108, 36–48.

Inman, A., Winter, M., Wheeler, R., Vrain, E., Lovett, A., Collins, A., Jones, I., Johnes, P., Cleasby, W., 2018. An exploration of individual, social and material factors influencing water pollution mitigation behaviours within the farming community. Land Use Policy 70, 16–26. https://doi.org/10.1016/j.landusepol.2017.09.042

IPCC, 2014. Climate Change 2014: Impacts, Adaptation, and Vulnerability. Summaries, Frequently Asked Questions, and Cross-Chapter Boxes. A Contribution of Working Group II to the Fifth Assessment Report of the Intergovernmental Panel on Climate Change [Field, C.B., V.R. Barros, D.J. Dokken, K.J. Mach, M.D. Mastrandrea, T.E. Bilir, M. Chatterjee, K.L. Ebi, Y.O. Estrada, R.C. Genova, B. Girma, E.S. Kissel, A.N. Levy, S. MacCracken,P.R. Mastrandrea, and L.L. White (eds.)] 190.

Kay, C., 2002. Chile’s neoliberal agrarian transformation and the peasantry. Journal of agrarian change 2, 464–501. https://doi.org/10.1111/1471-0366.00043

Kovács-Hostyánszki, A., Espíndola, A., Vanbergen, A.J., Settele, J., Kremen, C., Dicks, L.V., 2017. Ecological intensification to mitigate impacts of conventional intensive land use on pollinators and pollination. Ecology Letters 20, 673–689. https://doi.org/10.1111/ele.12762

Krause, R.W., Huisman, M., Steglich, C., Sniiders, T.A., 2018. Missing network data a comparison of different imputation methods, in: 2018 IEEE/ACM International Conference on Advances in Social Networks Analysis and Mining (ASONAM). IEEE, pp. 159–163.

Krause, R.W., Huisman, M., Steglich, C., Snijders, T., 2020. Missing data in cross-sectional networks–An extensive comparison of missing data treatment methods. Social Networks 62, 99–112. https://doi.org/10.1016/j.socnet.2020.02.004

Labarthe, P., Laurent, C., 2013. Privatization of agricultural extension services in the EU: Towards a lack of adequate knowledge for small-scale farms? Food Policy 38, 240–252. https://doi.org/10.1016/j.foodpol.2012.10.005

Lee, C.K., Kofman, Y., 2012. The politics of precarity: views beyond the United States. Work and Occupations 39, 388–408. https://doi.org/10.1177/0730888412446710

Lusher, D., Robins, G., 2013. Formation of social network structure. Exponential random graph models for social networks 16–28. Cambridge University Press.

Martín-López, B., Iniesta-Arandia, I., García-Llorente, M., Palomo, I., Casado-Arzuaga, I., Amo, D.G.D., Gómez-Baggethun, E., Oteros-Rozas, E., Palacios-Agundez, I., Willaarts, B., González, J.A., Santos-Martín, F., Onaindia, M., López-Santiago, C., Montes, C., 2012. Uncovering Ecosystem Service Bundles through Social Preferences. PLoS ONE 7, e38970. https://doi.org/10.1371/journal.pone.0038970

Martinez-Baron, D., Orjuela, G., Renzoni, G., Loboguerrero Rodríguez, A.M., Prager, S.D., 2018. Small-scale farmers in a 1.5°C future: The importance of local social dynamics as an enabling factor for implementation and scaling of climate-smart agriculture. Current Opinion in Environmental Sustainability, Sustainability governance and transformation 2018 31, 112–119. https://doi.org/10.1016/j.cosust.2018.02.013

Noticias de Navarra, 2017. Solo cuatro cultivos ocupan el 64% de las hectáreas del Canal. Diario de Noticias de Navarra [WWW Document]. URL http://www.noticiasdenavarra.com/2017/02/19/economia/solo-cuatro-cultivos-ocupan-el-64-de-las-hectareas-del-canal (accessed 5.16.17).

Organization, W.H., 2020. Coronavirus disease 2019 (COVID-19): situation report, 72.

O'Rourke, E., Charbonneau, M., Poinsot, Y., 2016. High nature value mountain farming systems in Europe: Case studies from the Atlantic Pyrenees, France and the Kerry Uplands, Ireland. Journal of Rural Studies 46, 47–59. https://doi.org/10.1016/j.jrurstud.2016.05.010

Palacios, D., Villalobos, C., 2016. Redes académicas al interior de las escuelas chilenas: Un estudio exploratorio utilizando Modelos Exponenciales de Grafos Aleatorios (ERGM). Redes. Revista hispana para el análisis de redes sociales 27, 33–44. http://dx.doi.org/10.5565/rev/redes.

Papadopoulos, A.G., Fratsea, L.-M., Mavrommatis, G., 2018. Governing migrant labour in an intensive agricultural area in Greece: Precarity, political mobilization and migrant agency in the fields of Manolada. Journal of Rural Studies 64, 200–209. https://doi.org/10.1016/j.jrurstud.2018.03.013

Pascual, U., Palomo, I., Adams, W., Chan, K., Daw, T., Garmendia, E., Gómez-Baggethun, E., Groot, R. de, Mace, G., Martin-Lopez, B., Phelps, J., 2017. Off-stage ecosystem service

burdens: A blind spot for global sustainability. Environ. Res. Lett. https://doi.org/10.1088/1748-9326/aa7392

Pellegrini, P., Fernández, R.J., 2018. Crop intensification, land use, and on-farm energy-use efficiency during the worldwide spread of the green revolution. PNAS 115, 2335–2340. https://doi.org/10.1073/pnas.1717072115

Plataforma URA Nueva Cultura, [WWW Document], 2014. URL https://www.bing.com/search?pc=COSP&ptag=D032820-N0610A915F698E57&form=CONBDF&conlogo=CT3335818&q=Plataforma%20URA%20Nueva%20Cultura%2C%202014 (accessed 4.11.20).

Plieninger, T., Bieling, C., 2013. Resilience-based perspectives to guiding high-nature-value farmland through socioeconomic change. Ecology and Society 18. http://dx.doi.org/10.5751/ES-05877-180420

Prell, C., Hubacek, K., Reed, M., 2009. Stakeholder analysis and social network analysis in natural resource management. Society and Natural Resources 22, 501–518. https://doi.org/10.1080/08941920802199202

Reyers, B., Nel, J.L., O'Farrell, P.J., Sitas, N., Nel, D.C., 2015. Navigating complexity through knowledge coproduction: Mainstreaming ecosystem services into disaster risk reduction. Proceedings of the National Academy of Sciences 112, 7362–7368. https://doi.org/10.1073/pnas.1414374112

Robins, G., Pattison, P., Kalish, Y., and Lusher, D. (2007). An introduction to exponential random graph models for social networks. Social Networks, 29(2):169-348. https://doi.org/10.1016/j.socnet.2006.08.002

Robins, G.L., Lusher, D., 2013. What are exponential random graph models (ERGM)? Lusher D., Koskinen J., Robins G.,(éds.), Exponential random graph models for social networks: Theory, methods, and applications. Cambridge University Press.

Schlüter, U., Mascher, M., Colmsee, C., Scholz, U., Bräutigam, A., Fahnenstich, H., Sonnewald, U., 2012. Maize source leaf adaptation to nitrogen deficiency affects not only nitrogen and carbon metabolism but also control of phosphate homeostasis. Plant Physiology 160, 1384–1406. https://doi.org/10.1104/pp.112.204420

Schmidt, K.A., Dall, S.R.X., Van Gils, J.A., 2010. The ecology of information: an overview on the ecological significance of making informed decisions. Oikos 119, 304–316. https://doi.org/10.1111/j.1600-0706.2009.17573.x

Siciliano, M.D., 2015. Advice Networks in Public Organizations: The Role of Structure, Internal Competition, and Individual Attributes. Public Administration Review 75, 548–559. https://doi.org/10.1111/puar.12362

Stensrud, A.B., 2019. Safe milk and risky quinoa: The lottery and precarity of farming in Peru. Focaal 2019, 72–84. https://doi.org/10.3167/fcl.2019.830108

Team, R.C., Bivand, R., Carey, V.J., DebRoy, S., Eglen, S., Guha, R., Herbrandt, S., Lewin-Koh, N., Myatt, M., Nelson, M., 2020. Package "foreign."

Thomas, M., Caillon, S., 2016. Effects of farmer social status and plant biocultural value on seed circulation networks in Vanuatu. Ecology and Society 21. http://dx.doi.org/10.5751/ES-08378-210213

Tilman, D., Balzer, C., Hill, J., Befort, B.L., 2011. Global food demand and the sustainable intensification of agriculture. Proceedings of the national academy of sciences 108, 20260–20264. http://dx.doi.org/10.1016/j.apsoil.2012.12.019

Vanwalleghem, T., Gómez, J.A., Amate, J.I., De Molina, M.G., Vanderlinden, K., Guzmán, G., Laguna, A., Giráldez, J.V., 2017. Impact of historical land use and soil management change on soil erosion and agricultural sustainability during the Anthropocene. Anthropocene 17, 13–29. https://doi.org/10.1016/j.ancene.2017.01.002

Villamayor-Tomas, S., Sagebiel, J., Olschewski, R., 2019. Bringing the neighbors in: A choice experiment on the influence of coordination and social norms on farmers' willingness to accept agro-environmental schemes across Europe. Land use policy 84, 200–215. https://doi.org/10.1016/j.landusepol.2019.03.006

Wasserman, S., Pattison, P., 1996. Logit models and logistic regressions for social networks: I. An introduction to Markov graphs andp. Psychometrika 61, 401–425.

Wilby, A., Thomas, M.B., 2002. Natural enemy diversity and pest control: patterns of pest emergence with agricultural intensification. Ecology Letters 5, 353–360. https://doi.org/10.1046/j.1461-0248.2002.00331.x

Zhang, W., Ricketts, T.H., Kremen, C., Carney, K., Swinton, S.M., 2007. Ecosystem services and dis-services to agriculture. Ecol.Econ. 64, 253–260. https://doi.org/10.1046/j.1461-0248.2002.00331.x

Figure
Click here to access/download;Figure;Figure1(1).tif

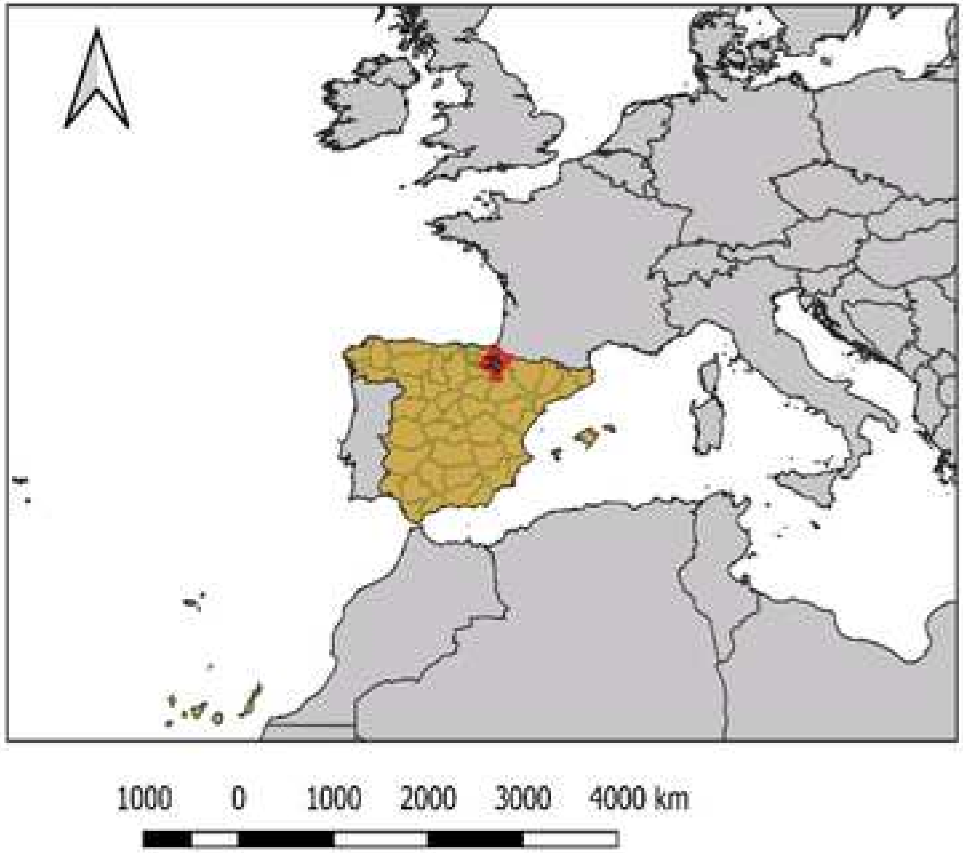

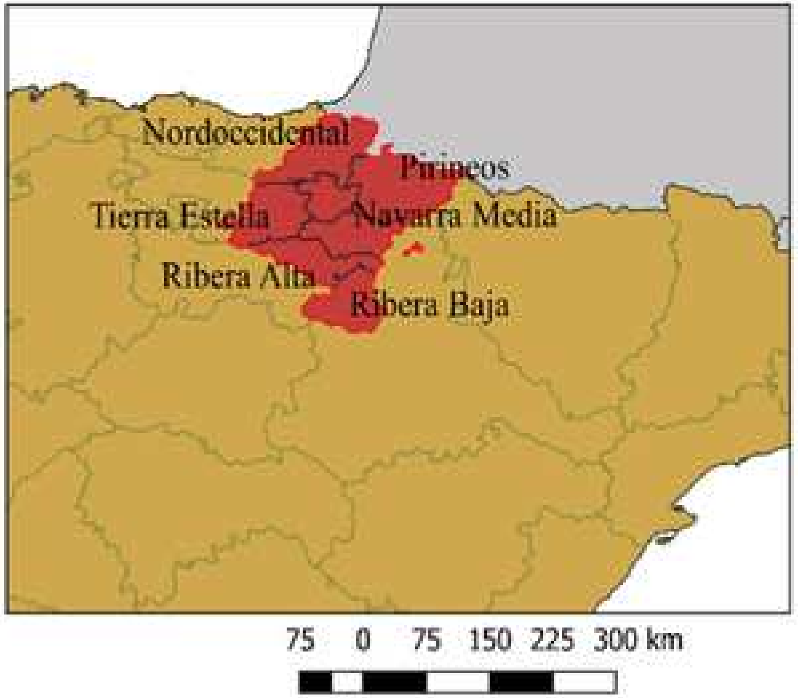

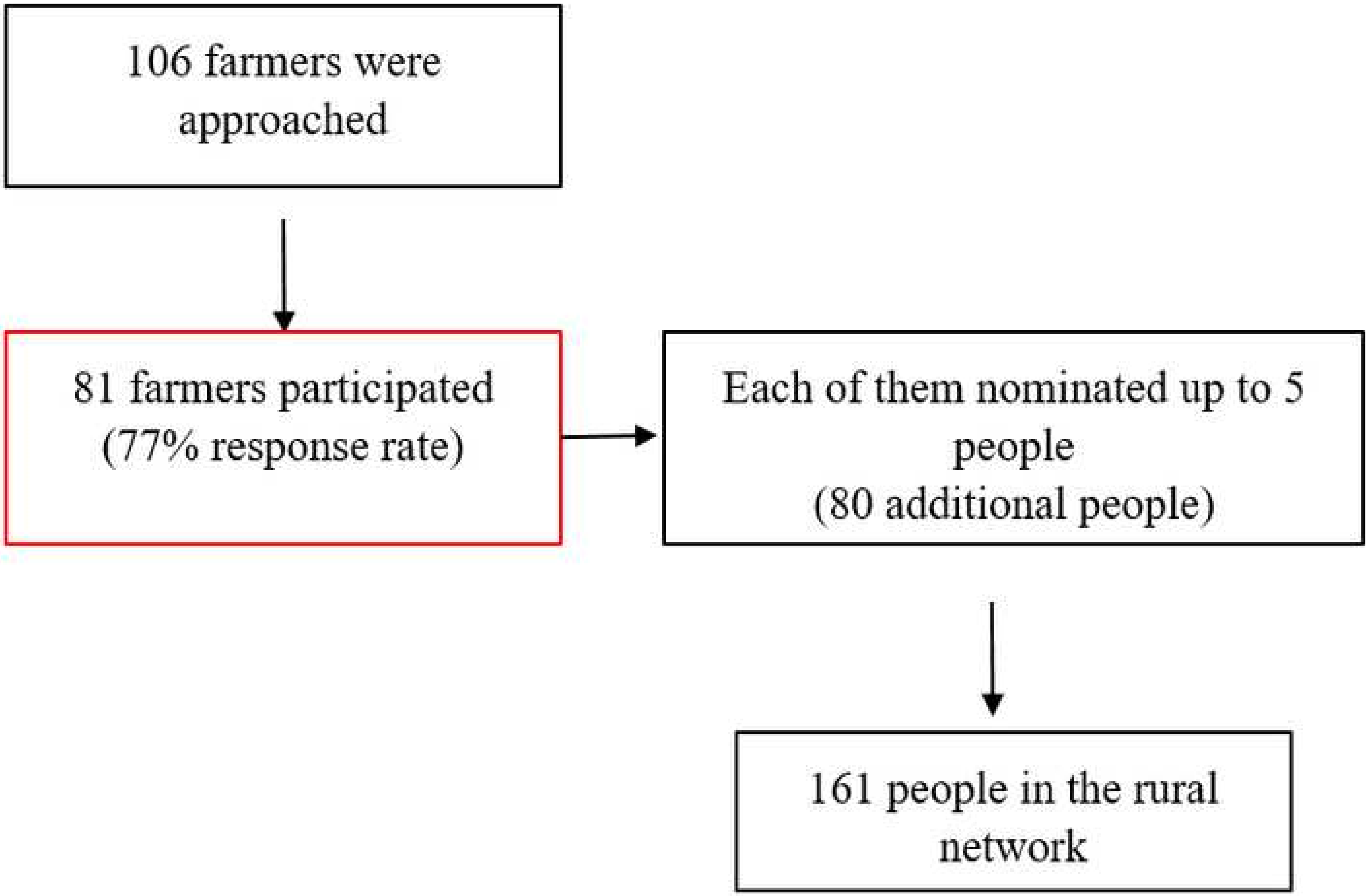
106 farmers were approached
81 farmers participated (77% response rate)
Each of them nominated up to 5 people (80 additional people)
161 people in the rural network

Figure
Click here to access/download;Figure;Figure3(1).tif

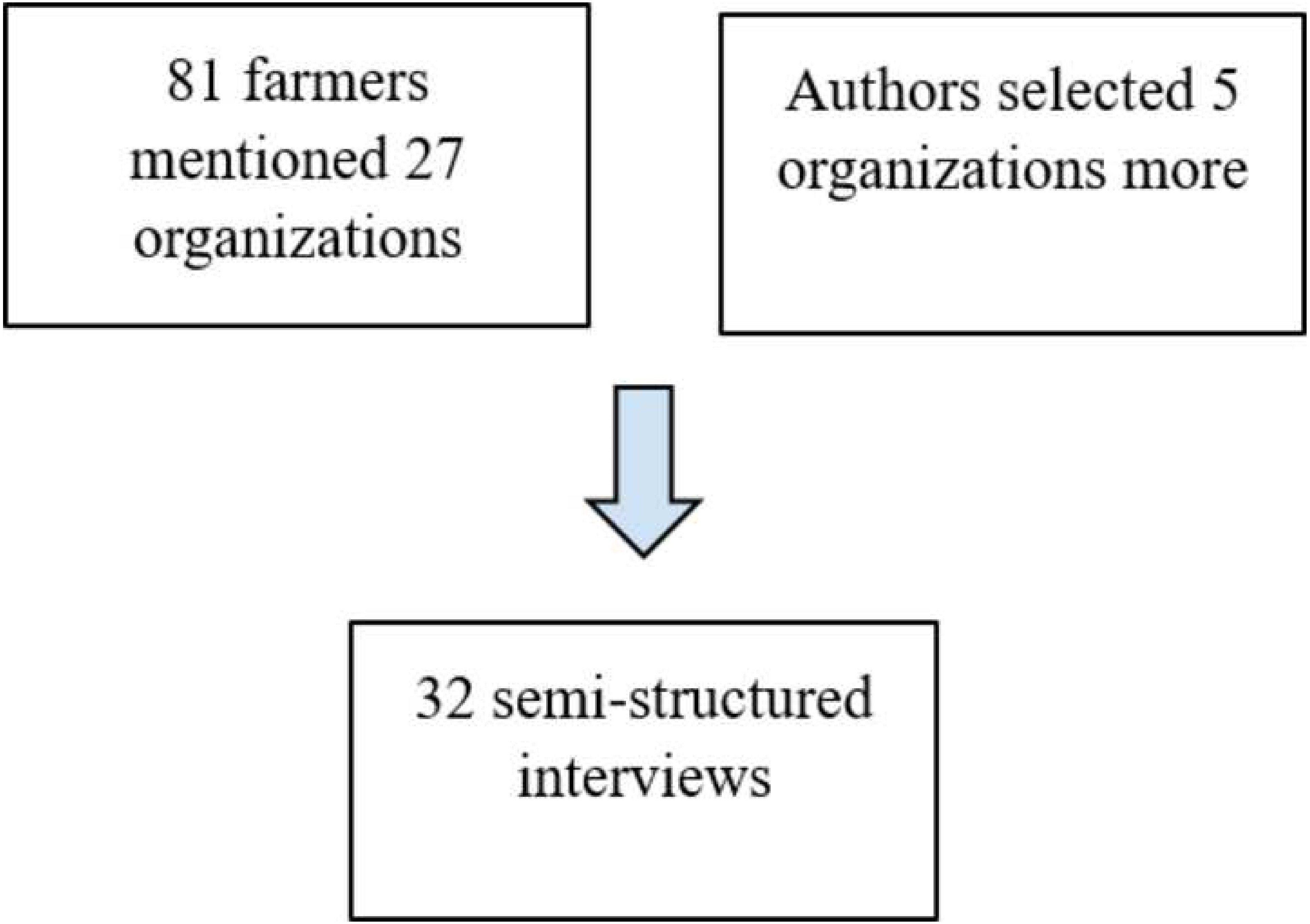

Figure

Click here to access/download;Figure;Figure4(1).tif

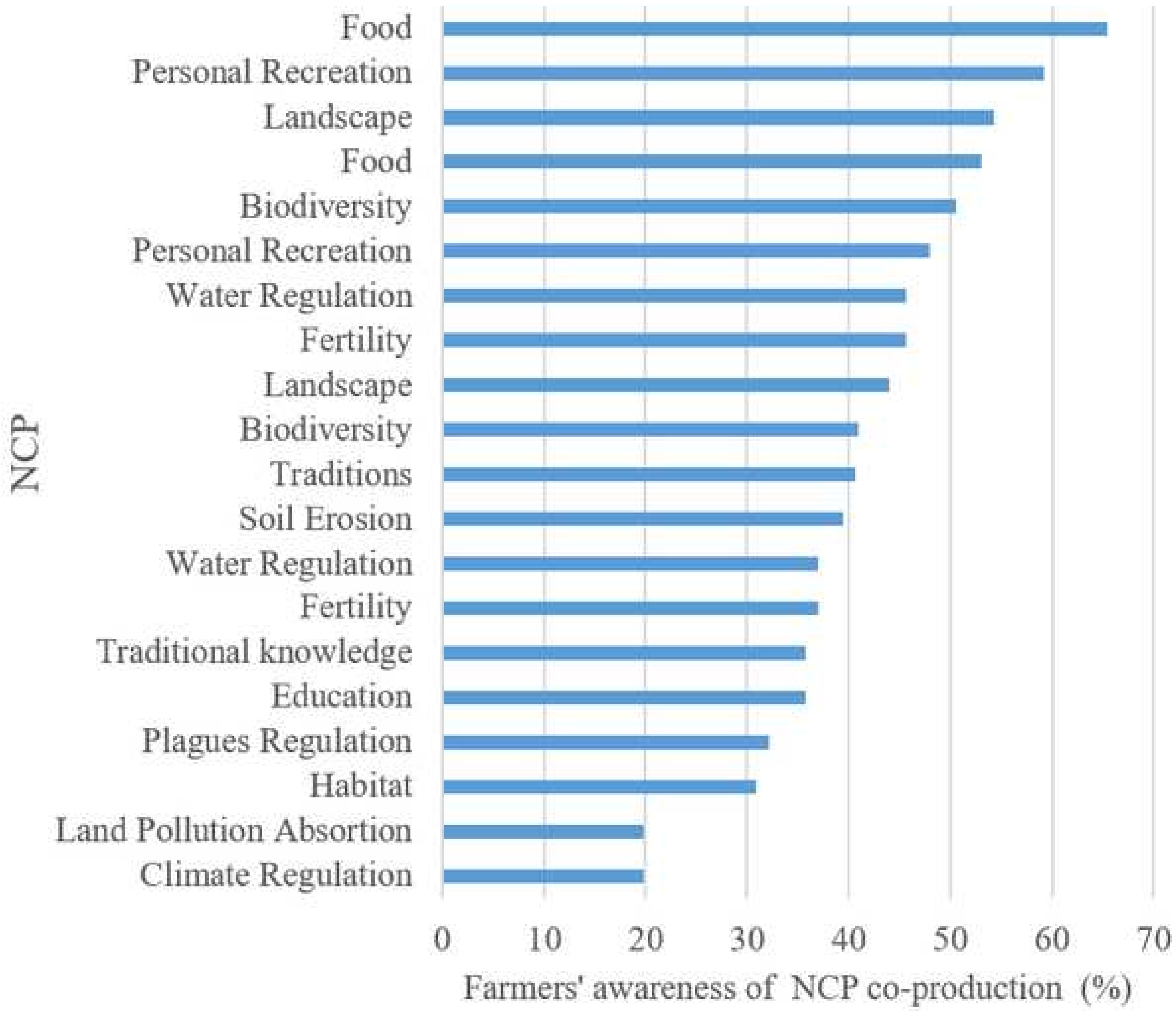

Click here to access/download;Figure;Figure5(1).tif

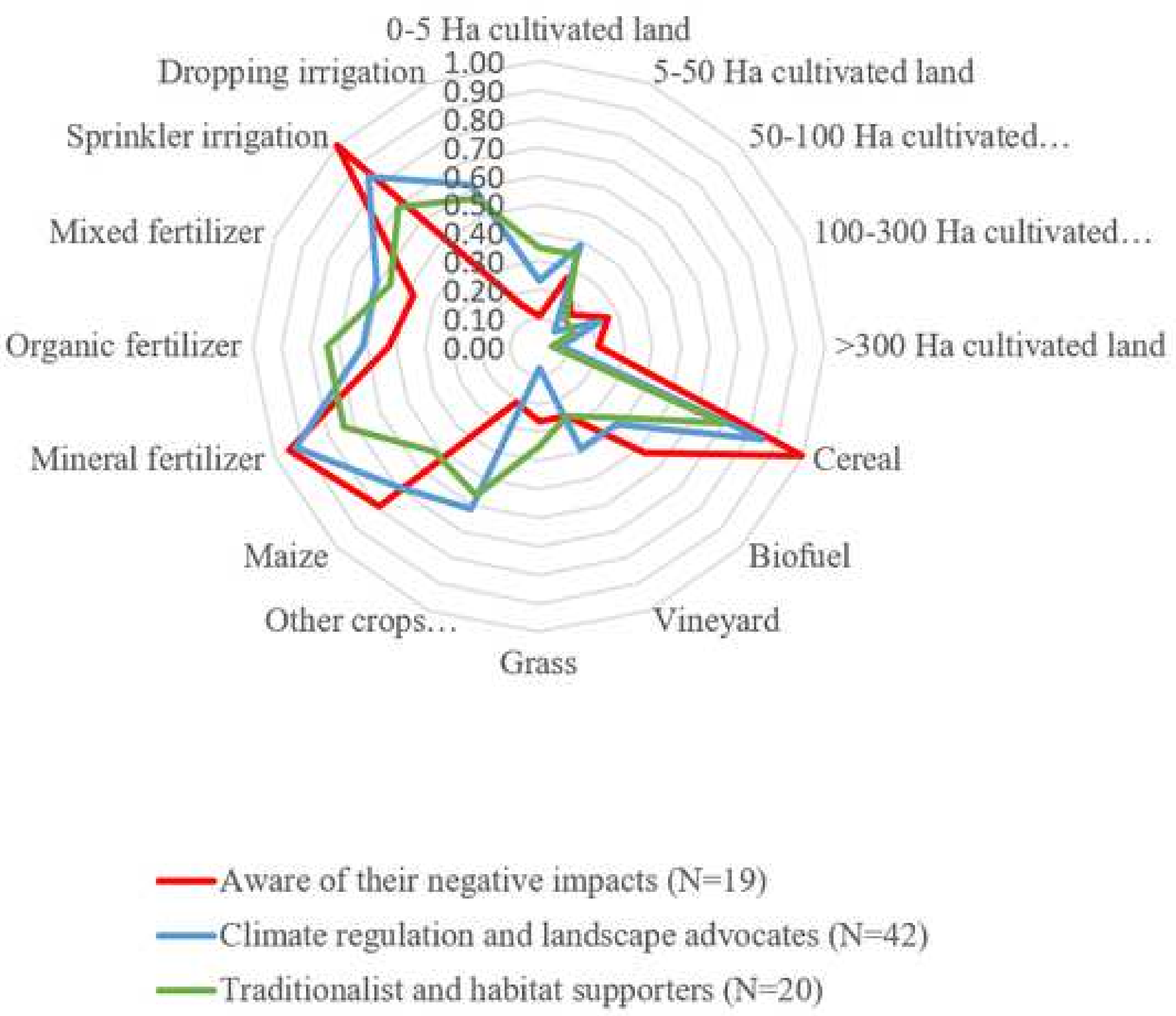

Click here to access/download;Figure;Figure6(1).tif

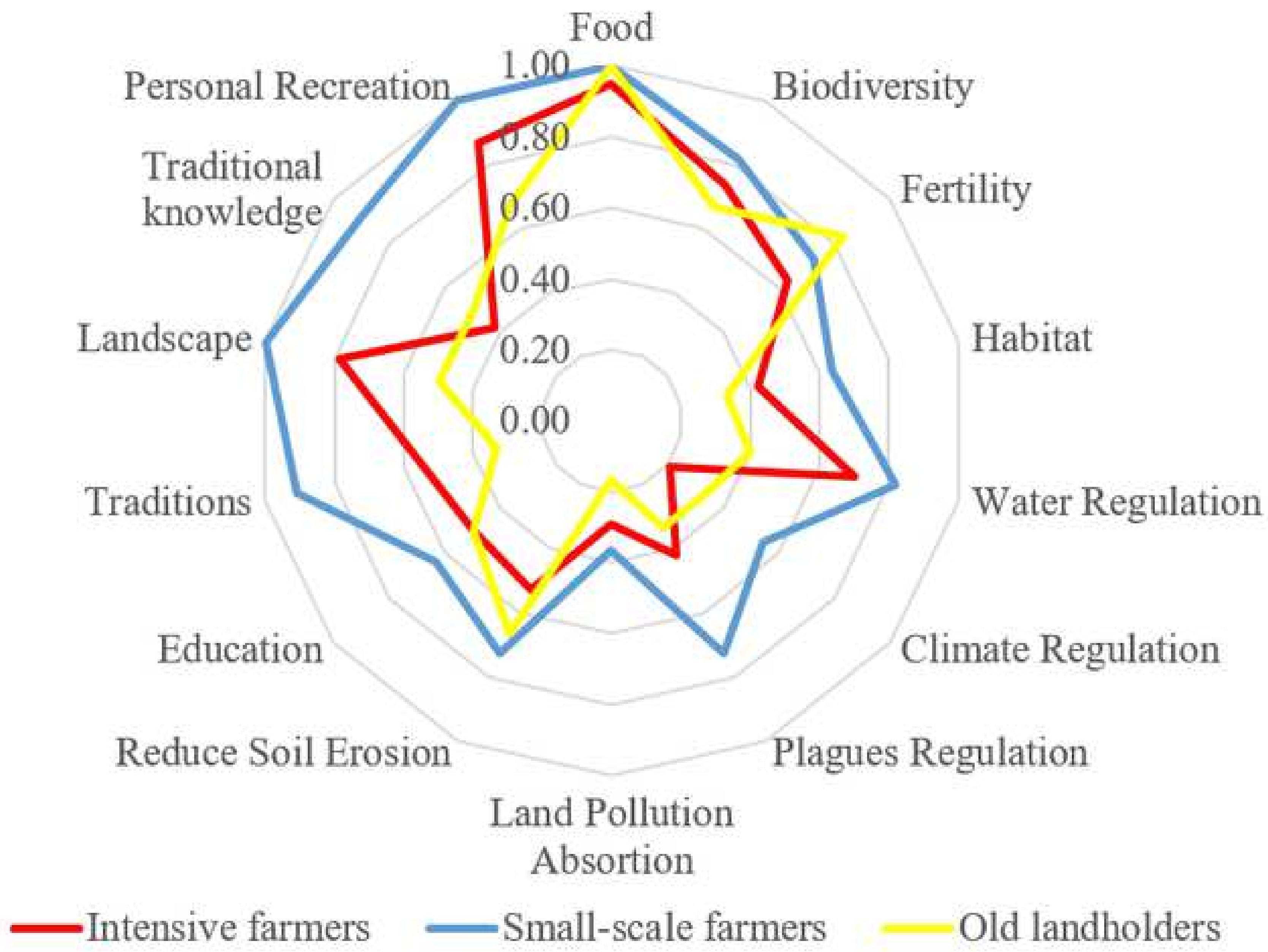

Figure

Click here to access/download;Figure;Figure7(1).tif

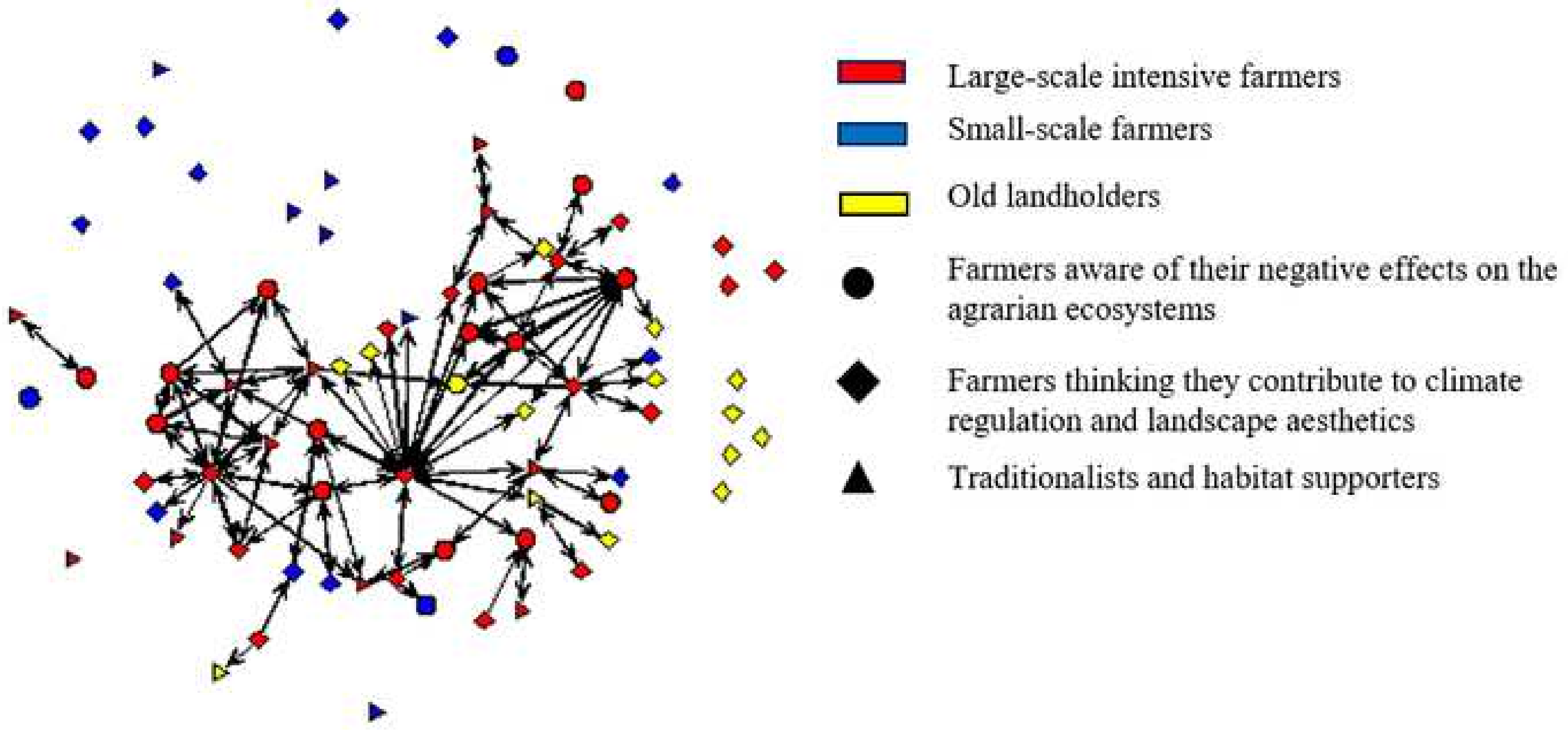

Click here to access/download
Supporting Information
Supplementary material.docx
